# Phenotyping Preeclampsia Using Unsupervised Machine Learning: A Prospective Cohort Study

**DOI:** 10.1111/1471-0528.70262

**Published:** 2026-05-14

**Authors:** Ohad Houri, Lina Youssef, Francesca Crovetto, Maria Borrell, Maddalena Crimella, Maria Giulia Ferrante, Rommy H. Novoa, Irene Casas, Noelia Encabo, Leticia Benitez, Marta Larroya, Anna Peguero, Eva Meler, Sara Castro‐Barquero, Bart Bijnens, Francesc Figueras, Eduard Gratacos, Gabriel Bernardino, Fàtima Crispi

**Affiliations:** ^1^ BCNatal—Barcelona Center for Maternal‐Fetal and Neonatal Medicine (Hospital Clínic and Hospital Sant Joan de Deu), IDIBAPS University of Barcelona Barcelona Spain; ^2^ Primary Care Interventions to Prevent Maternal and Child Chronic Diseases of Perinatal and Developmental Origin RD21/0012/0003 Instituto de Salud Carlos III Madrid Spain; ^3^ Dexeus Mujer (Hospital Universitari Dexeus) Barcelona Spain; ^4^ Centro de Investigación Biomédica en Red de Fisiopatología de la Obesidad y Nutrición (CIBEROBN) Madrid Spain; ^5^ BCN Medtech, Department of Information and Communication Technologies Universitat Pompeu Fabra Barcelona Spain; ^6^ ICREA Barcelona Spain; ^7^ Centre for Biomedical Research on Rare Diseases (CIBERER) Barcelona Spain

**Keywords:** cluster analysis, preeclampsia, pregnancy complications, unsupervised machine learning

## Abstract

**Objective:**

To explore clinically meaningful phenotypes of preeclampsia using unsupervised machine learning.

**Design:**

Prospective cohort study.

**Setting:**

BCNatal, a tertiary maternal‐foetal medicine centre (Barcelona, Spain).

**Population:**

A total of 482 pregnant women diagnosed with preeclampsia between August 2013 and April 2024.

**Methods:**

Maternal demographic, clinical, ultrasound and laboratory data were prospectively collected, together with delivery details and maternal and neonatal complications. We generated a patient representation using maternal age, height, weight, body‐mass index, blood pressure, angiogenic factors, urinary albumin/creatinine ratio, gestational age at birth and birthweight centile. Dimensionality reduction was performed using Uniform Manifold Approximation and Projection, followed by *k*‐means clustering to identify phenotypes.

**Main Outcome Measures:**

Maternal and neonatal characteristics and complication rates were compared across clusters to evaluate the clinical significance of the data‐driven phenotypes.

**Results:**

Three phenotypes were identified. Cluster A (*n* = 223; 46.2%) showed earlier delivery (mean 33.1 ± SD 3.3 weeks), marked angiogenic imbalance, prevalent foetal growth restriction (64%) and the highest rates of maternal (23%) and neonatal (41%) complications. Cluster B (*n* = 147; 30.5%) had later delivery (37.0 ± 2.1 weeks), moderate angiogenic imbalance and intermediate birthweight centiles (15.5 ± 9.7). Cluster C (*n* = 112; 23.2%) comprised mostly term cases (38.2 ± 1.5 weeks) with the lowest angiogenic imbalance and the highest birthweight centile (67.9 ± 27.3); obesity (31%) and diabetes (15%) were most prevalent and maternal (4%) and neonatal (9%) complications were least frequent.

**Conclusions:**

Unsupervised learning delineated three preeclampsia phenotypes. These phenotypes could support the need for future risk stratification and more personalised management; prospective external validation is warranted.

## 1 Introduction

Preeclampsia is a complex, multisystem endothelial disorder affecting 3%–7% of pregnancies and remains a leading contributor to maternal morbidity and mortality worldwide [1, 2, 3, 4, 5, 6, 7]. Despite its recognised clinical and pathophysiological heterogeneity, preeclampsia is still predominantly classified based on the severity of its end‐stage clinical manifestations [8, 9, 10, 11, 12, 13]. While this stratification is useful for anticipating adverse outcomes, it hampers the integration of the diverse pathophysiological pathways into targeted strategies for prevention and management [8, 12, 14, 15]. Over the past two decades, accumulating evidence has progressively delineated two main phenotypes of preeclampsia: early‐onset preeclampsia, characterised by placental insufficiency and foetal growth restriction, versus late preeclampsia, typically with lower frequency of placental insufficiency and foetal growth restriction [1, 3, 16, 17, 18]. According to this view, early preeclampsia would be primarily driven by poor placental implantation while late cases may result from suboptimal maternal cardiovascular adaptation to pregnancy [14, 19, 20, 21, 22]. Although this framework represents a remarkable advancement in understanding the disease, it still does not fully capture the clinical spectrum. For instance, a proportion of late‐onset cases present features of placental insufficiency and foetal growth restriction, underscoring the limitations of the above dichotomy [23, 24]. More recently, a new classification system has been proposed based on maternal hemodynamics and foetal growth: one phenotype associated with foetal growth restriction characterised by vascular resistance and another phenotype by normal foetal growth and intravascular‐volume induced hypertension [18]. There remains, therefore, a need to refine current pathophysiological subgroups, to better reflect the clinical and pathophysiological diversity of the preeclamptic syndrome [8, 12, 14, 16, 25]. However, advancing these goals through conventional clinical research is challenging. Despite extensive evidence supporting the heterogeneity of etiopathogenic factors and clinical forms, most studies focus on isolated domains, such as gestational age at onset, maternal cardiometabolic profile, placental involvement or the association with small for gestational age (SGA) neonates [3, 8, 9, 16, 17, 19, 20, 21, 24]. To date, there have been limited efforts to integrate these domains within a unified analytical framework. Emerging interpretable machine learning (ML) methodologies offer a promising avenue to overcome this limitation, facilitating the integration of heterogeneous data sources and potentially enabling a more nuanced understanding of the phenotypes of preeclampsia [26, 27].

Recent advancements in ML have provided powerful tools for analysing complex datasets through a comprehensive integration of clinical, laboratory and ultrasonographic data, allowing identification of hidden patterns within heterogeneous populations [28, 29, 30]. ML has been pivotal to generate personalised targeted prediction and prevention of cardiovascular disease in adults, including the identification of phenotypes of heart failure, coronary artery disease or heart transplant outcomes [29, 31, 32, 33, 34, 35]. By grouping similar patients together in multiple dimensions, it is then possible to analyse the characteristics of similarly grouped individuals and relate them to complications or therapeutic responses. Clustering techniques have been successfully applied to other obstetric conditions such as SGA or gestational diabetes mellitus, uncovering phenotypes with unique characteristics and tailored treatment needs [32, 34, 35, 36, 37]. Preeclampsia represents a paradigm for the use of advanced data mining techniques to boost improved definitions that recognise better individual variability.

This study aimed to explore data‐driven phenotypes of preeclampsia using unsupervised ML and to evaluate how these relate to clinically recognised patterns of disease.

## 2 Methods

### 2.1 Study Population and Design

A prospective cohort comprising women diagnosed with preeclampsia attended at BCNatal (Hospitals Clínic and Sant Joan de Déu, Barcelona, Spain) from August 2013 to April 2024. Preeclampsia was defined as the onset of hypertension after 20 weeks of gestation accompanied by proteinuria or end‐organ damage [1, 38].

### 2.2 Data Collection and Study Protocol

The study involved the collection of maternal demographics, pregnancy and neonatal data from electronic hospital databases.

First‐trimester screening data, such as maternal blood pressure and uterine arteries mean pulsatility index (PI), were retrieved from the medical records.

At preeclampsia, maternal clinical, laboratory and ultrasonographic data were collected. Maternal serum concentrations of placental growth factor (PlGF) and soluble fms‐like tyrosine kinase‐1 (sFlt1) were measured using an automated immunoassay platform (Atellica IM Analyser, Siemens). All participants underwent feto‐placental ultrasound. Estimated foetal weight was calculated using the Hadlock formula [39] and centile based on local reference curves [40]. Feto‐placental Doppler included uterine, umbilical artery (UA), middle cerebral arteries (MCAs) and ductus venosus [41, 42, 43]. Cerebroplacental ratio was calculated as MCA‐PI/UA‐PI [44].

At the time of delivery, occurrence of SGA, severe SGA and large‐for‐gestational‐age (LGA) neonates were defined as birthweight below the 10th or 3rd centile or above the 90th centile respectively, according to local charts [40]. Severe maternal complications were defined as the presence of HELLP syndrome, central nervous system or renal dysfunction, pulmonary oedema, requirement for transfusion or maternal death. Severe neonatal complications were defined as the occurrence of need for resuscitation or the presence of neonatal complications such as respiratory distress syndrome, pulmonary hypoplasia, necrotizing enterocolitis, neonatal infection and brain intraventricular haemorrhage or death.

### 2.3 Clustering Algorithm

**FIGURE 1:**
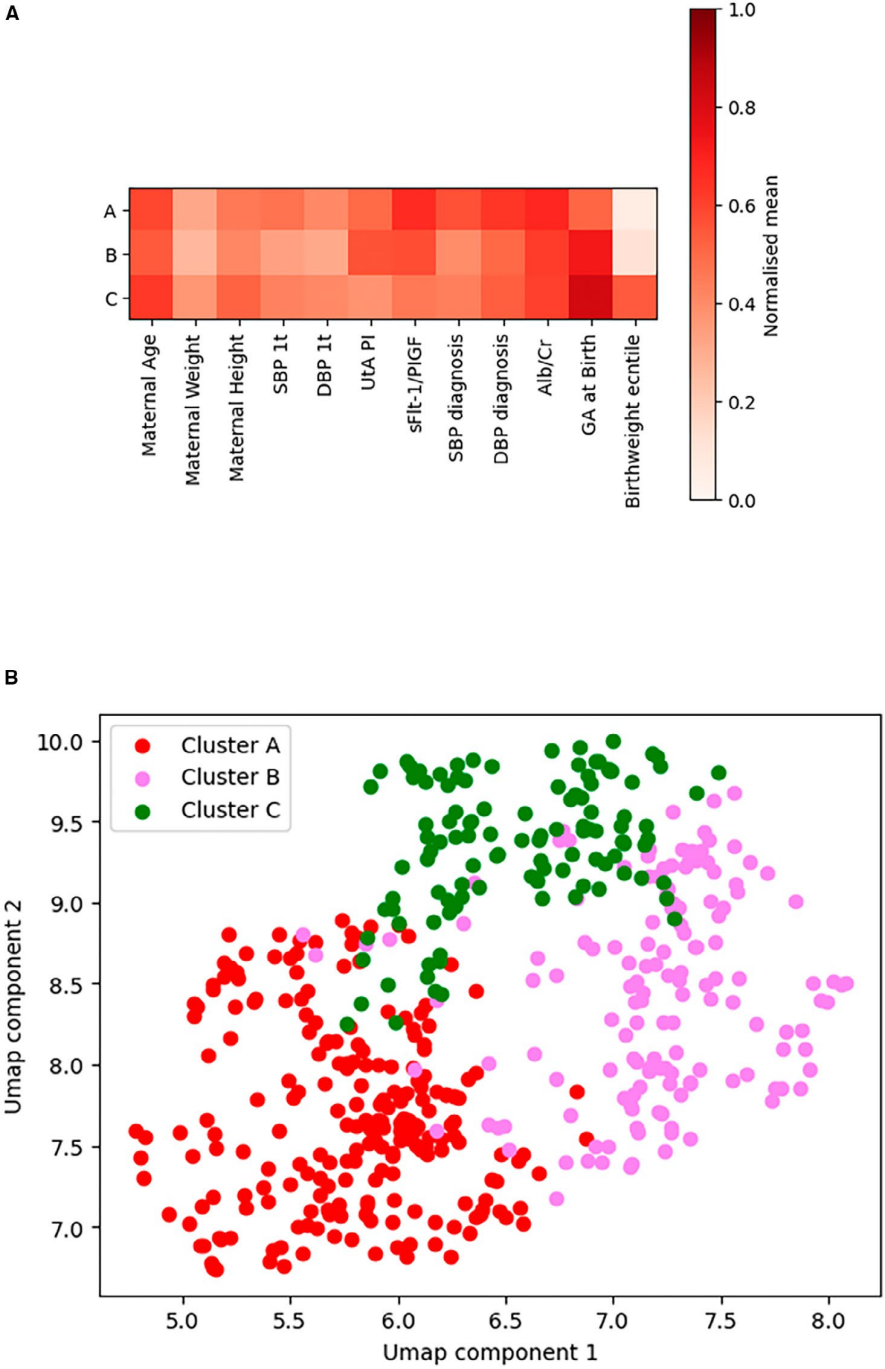
(A) Heatmap of the parameters used for preeclampsia clustering. The heatmap displays the normalised mean values of the parameters used to cluster preeclampsia cases into three distinct phenotypes (A, B and C). (B) Scatter plot of the reduced space combining all feto‐maternal characteristics used for the clustering, coloured according to the assigned cluster. Each point represents an individual preeclampsia, projected from the high‐dimensional feature space into two dimensions. Colours indicate cluster membership as assigned by the *K*‐means algorithm, with three distinct clusters identified. Cluster A depicted in red, Cluster B in violet and Cluster C in green. 1 t, first trimester; Alb/cr, urinary albumin/creatinin ratio; BMI, body mass index; DBP, diastolic blood pressure; GA, gestational age; PlGF, placental growth factor; SBP, systolic blood pressure; sFlt1, soluble fms‐like tyrosine kinase 1; UMAP, uniform manifold approximation and projection.

To identify and visualise phenotypes within the preeclampsia cohort, we employed a two‐step process involving dimensionality reduction and clustering using Python (version 3.12.4). The prespecified variables for the clustering analyses were maternal age, height, weight and body mass index (BMI), sFlt1/PlGF ratio, systolic and diastolic blood pressure and urinary albumin/creatinine at the time of diagnosis, gestational age at birth and birthweight centile (Figure 1A). These variables were selected based on their clinical relevance and were restricted to continuous variables to facilitate scaling and avoid potential overrepresentation of categorical variables in the clustering process.

First, all variables were standardised using *z*‐score normalisation to avoid their different ranges and scales. Next, a reduced representation of the prespecified variables was obtained by Uniform Manifold Approximation and Projection (UMAP) (5 components, random state 42) [45]. Second, the *K*‐Means algorithm was used to partition the population into clusters according to their values in the UMAP representation (*k* = 3, random state 1). Robustness was assessed through sensitivity analyses of UMAP hyperparameters and repeated clustering on random subsamples (Supporting Information). The optimal number of clusters was automatically selected to maximise the silhouette criteria [46]. To avoid the algorithm to only the already established partition in late and early preeclampsia, we set the minimum number of clusters to 3. An increasing number of clusters was tested until clusters with less than 50 individuals appeared, which we deemed too few. To assess clustering stability, we repeated the dimensionality reduction and clustering procedure 10 times. In all 10 repetitions, the optimal number of clusters remained 3% and 97% of samples were assigned to the same cluster across runs. Finally, traditional statistical analyses were conducted to compare the maternal, pregnancy and neonatal characteristics of the preeclampsia clusters identified by ML.

Categorical variables were summarised as counts and percentages, and continuous variables as means with standard deviations or medians with interquartile ranges, depending on distribution. Differences between clusters were assessed using one‐way ANOVA for normally distributed continuous variables and the Kruskal–Wallis test for non‐normally distributed variables. Categorical variables were compared using the *χ* ^2^ test or Fisher's exact test when appropriate. Statistical significance was set at *p* < 0.05.

### 2.4 Ethics Approval

The study was approved by the Institutional Review Board (HCB2014/7154 and PIC‐100‐24). All patients provided written informed consent.

## 3 Results

### 3.1 ML Identifies Three Phenotypes of Preeclampsia

From the initial 538 eligible preeclampsia cases, 56 could not be included due to missing data necessary for the clustering, resulting in a final analytic sample of 482 pregnancies (Figure S1). The characteristics of the study population are shown in Tables S1–S7.

ML determined that the optimal number of preeclampsia clusters was three (Figure 1): Cluster A (*n* = 223, 46.3%), Cluster B (*n* = 147, 30.5%) and Cluster C (*n* = 112, 23.2%).

#### 3.1.1 Cluster A: Early Preeclampsia

**FIGURE 2:**
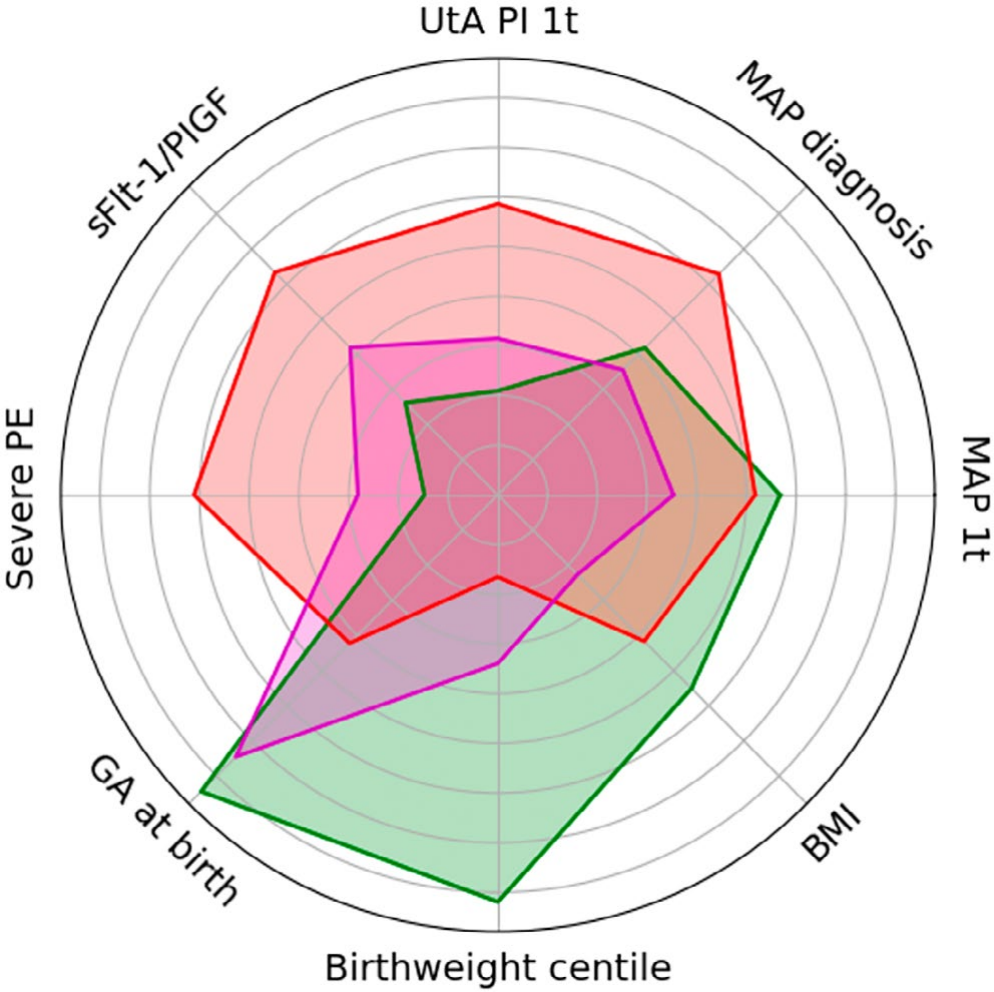
Radar plot representing key clinical parameters defining each preeclampsia phenotype. Cluster A depicted in red, Cluster B in violet and Cluster C in green. Feature values were normalised to a 0–1 scale based on mean values within the study population to enable visual comparison across variables with different units and scales. 1 t, first trimester; BMI, body mass index; GA, gestational age; MAP, mean arterial blood pressure; PE, preeclampsia; PlGF, placental growth factor; sFlt1, soluble fms‐like tyrosine kinase 1; UtA PI, uterine arteries mean PI.

**FIGURE 3:**
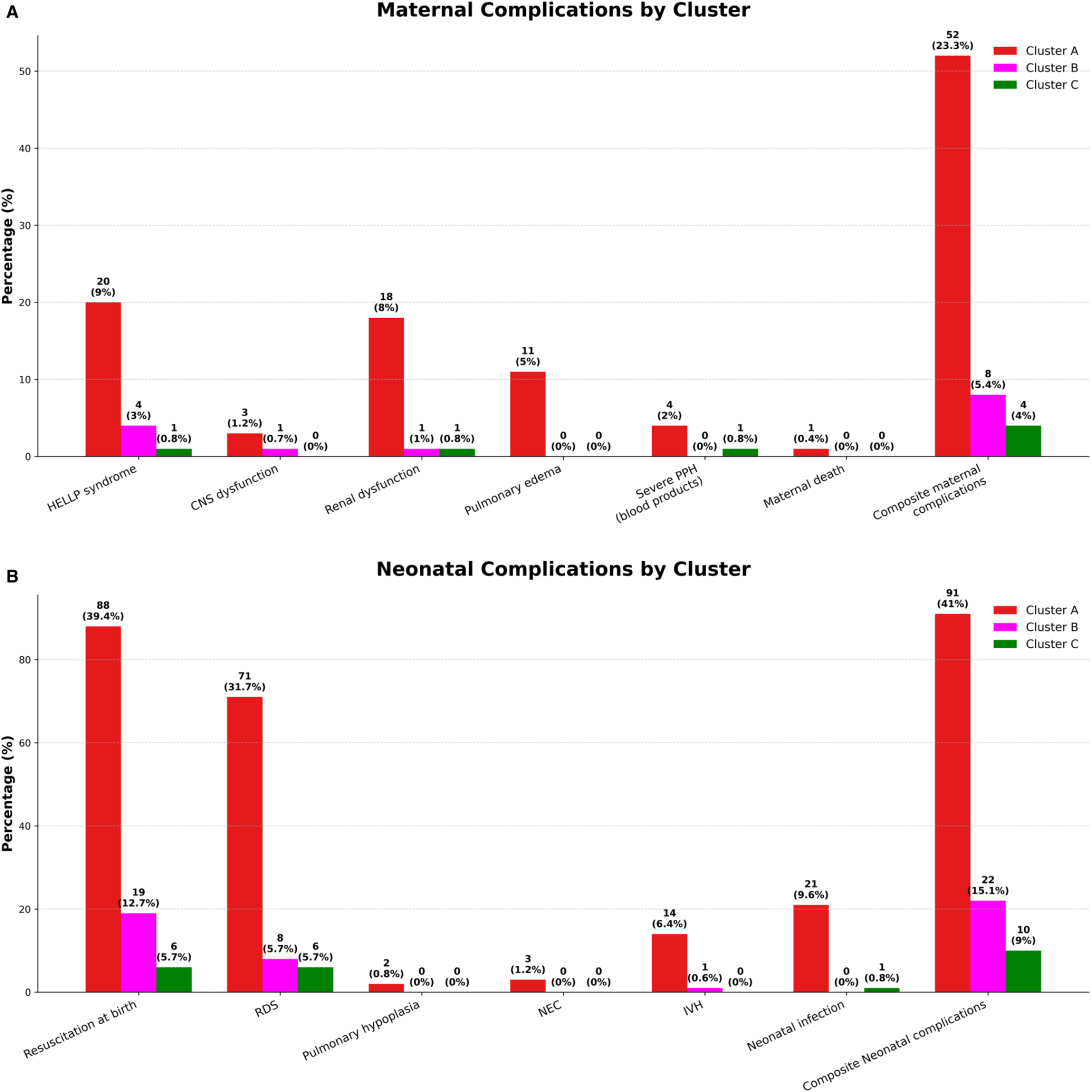
Maternal (A) and neonatal (B) complications according to the preeclampsia phenotypes. Severe maternal complications defined as the presence of any of the following: (i) HELLP syndrome (lactate dehydrogenase > 700 IU/L, aspartate aminotransferase to twice normal values and platelet count < 100 × 10^9^/L); (ii) central nervous system (CNS) dysfunction (eclampsia, stroke, reversible ischemic neurological deficit or cortical blindness); (iii) renal dysfunction dialysis, serum creatinine concentration greater than 150 μmol/L or urine output < 0.5 mL/kg/h during 12 h; or need for treatment with furosemide to maintain urine output > 0.5 mL/kg/h for 3 h; (v) pulmonary oedema; (vi) postpartum haemorrhage (PPH) with a requirement for transfusion of blood products red cells or (vii) maternal death. Severe neonatal complications defined as the occurrence of any of the following: (i) need for resuscitation at birth (including stimulation, continuous positive airway pressure, positive pressure ventilation, cardiopulmonary resuscitation or endotracheal intubation); (ii) or the presence of neonatal complications such as respiratory distress syndrome (RDS), pulmonary hypoplasia, necrotizing enterocolitis (NEC), neonatal infection, brain intraventricular haemorrhage (IVH) or death.

Cluster A was characterised by the lowest gestational age at delivery, greatest severity and highest angiogenic imbalance and association to SGA (Figures 2 and S2). As compared to the other clusters, Cluster A had the highest prevalence of previous hypertension (16%) (Table 1). At diagnosis, it presented the highest maternal blood pressure, uterine artery Doppler, sFlt1/PlGF, liver enzymes, uric acid, lactate dehydrogenase and urinary albumin/creatinine values (Table 1). Women from Cluster A required the most use of antihypertensive drugs (72%), magnesium sulphate (83%) and furosemide (11%) and showed the highest incidence of maternal severe complications (23%) with neurological signs (29%), HELLP syndrome (9%), kidney impairment (8%) and pulmonary oedema (5%), including the only two cases of eclampsia and one case of maternal death (Figure 3). Cluster A also showed the lowest gestational age at diagnosis (mean 32 weeks) and delivery (mean 33 weeks), and the highest rate of administration of corticosteroids (72%) for foetal lung maturation. Likewise, it was highly associated to SGA (83%) and severe SGA (68%) neonates (1C), as well as need for planned delivery (95%) and emergency caesarean section (33.7%). Neonates had the highest rate of complications (41%) and mortality (18%), with a mean stay of 37 days at NICU (Table 1 and Figure 3).

**TABLE 1:** Maternal characteristics across the preeclampsia phenotypes.

	Cluster A (*n* = 223)	Cluster B (*n* = 147)	Cluster C (*n* = 112)	*p*
Age, years	34.35 ± 5.48	34.26 ± 5.86	34.66 ± 5.36	0.8
Self‐reported ethnicity				
White	133 (56.7)	106 (64.6)	75 (56)	0.06
Hispanic	58 (23)	38 (22)	25 (24)	0.16
Asian	16 (6.7)	17 (8.1)	11 (12.4)	0.04
Low socioeconomic status^a^	89 (36)	46 (28)	51 (42)	0.07
Weight, kg	67.32 ± 12.91	59.13 ± 10.03	76.15 ± 17.6	< 0.01
Pregestational body mass index, kg/m^2^	25.72 ± 4.82	22.90 ± 3.31	28.66 ± 6.21	< 0.01
Comorbidities	89 (36)	29 (17)	74 (61)	< 0.01
Obesity^b^	53 (21.4)	30 (18)	38 (31)	< 0.01
Chronic hypertension	43 (17)	13 (7)	18 (14)	0.02
Pregestational diabetes mellitus	7 (4)	5 (2)	18 (15.4)	< 0.01
Autoimmune diseases	7 (2)	8 (5.4)	16 (11.8)	< 0.01
Renal disease	3 (1)	6 (3)	7 (5)	0.05
Previous preeclampsia	20 (16.3)	25 (15)	37 (15)	0.95
Smoking	32 (12)	19 (14.2)	6 (4)	0.06
Nulliparity	166 (67)	120 (73)	75 (62)	0.2
Use of assisted reproductive technologies	42 (17)	31 (19)	18 (15)	0.78
Maternal first trimester data				
Systolic blood pressure, mmHg	122.1 ± 12.2	114.6 ± 11.3	121.8 ± 11.6	< 0.01
Diastolic blood pressure, mmHg	77.4 ± 9.8	75.4 ± 9.8	79.7 ± 9.7	< 0.01
Uterine arteries mean PI	1.45 ± 0.54	1.0 ± 0.39	0.89 ± 0.51	< 0.01
Maternal data at diagnosis				
Gestational age at diagnosis, weeks	31.9 ± 3.9	36.6 ± 2.5	37.6 ± 1.7	< 0.01
Systolic blood pressure, mmHg	157.9 ± 18.0	141.0 ± 15.1	144.8 ± 11.9	< 0.01
Diastolic blood pressure, mmHg	98.8 ± 10.1	89.0 ± 9.1	92.7 ± 9.5	< 0.01
sFlt1/PlGF	3141 [219–3360]	561 [741–84]	213 [23–237]	< 0.01
Aspartate aminotransferase, U/L	42.65 [19–36]	36.14 [17–28]	22.0 [16–26]	< 0.01
Alanine aminotransferase, U/L	45.89 [17–38]	37.01 [14–27]	19.21 [13–22]	0.03
Uric acid, mg/dL	5.74 [4.8–6.7]	5.48 [5.6–6.13]	5.04 [4.25–5.7]	< 0.01
Lactate dehydrogenase, U/L	419 [267–470]	334 [215–441]	337 [223–406]	< 0.01
Platelet count, ×10^3^/L	198 [158–251]	175 [124–234]	219 [160–283]	< 0.01
Urinary albumin/creatinine	2603 [404–3720]	1070 [319–1194]	835 [327–785]	< 0.01
Use of antihypertensive drugs	175 (72)	56 (31)	46 (39)	< 0.01
Use of magnesium sulphate	208 (83)	59 (31)	21 (22)	< 0.01
Use of furosemide	29 (11)	4 (2)	2 (1)	< 0.01
Administration of corticosteroids for foetal lung maturation	180 (72)	30 (22)	7 (6)	< 0.01

*Note:* Data are presented as number (%) or mean ± standard deviation as appropriate.

Abbreviations: PI, pulsatility index; PlGF, placental growth factor; sFlt1 denotes soluble fms‐like tyrosine kinase 1.

^a^ Low socioeconomic status defined as no formal education beyond primary school and/or reported unemployment.

^b^ Obesity defined as body mass index above 30.

#### 3.1.2 Cluster B: Late Placental Preeclampsia

Cluster B was characterised by late gestational age at delivery (mean 37 weeks), the lowest maternal BMI and blood pressure and intermediate values of angiogenic profile, uterine artery Doppler and birthweight centile (Figures 2 and S2). As compared to the other clusters, Cluster B showed the lowest maternal weight, BMI (mean 22.9 kg/m^2^), blood pressure and rate of comorbidities (17%) (Table 1). It was associated with intermediate levels of angiogenic factors, liver enzymes, uric acid, lactate dehydrogenase, platelets, urinary albumin/creatinine and feto‐placental Doppler (Table 2). Association of severe maternal complications was low (5%). Mean gestational age at diagnosis and delivery of Cluster B were 36 and 37 weeks, respectively. Fifty four percent of the neonates were SGA and 31% severe SGA, with 9% of neonatal complications, 4% perinatal mortality and mean of 28 days of NICU stay (Table 2 and Figure 3).

**TABLE 2:** Pregnancy and neonatal characteristics across the preeclampsia phenotypes.

	Cluster A (*n* = 223)	Cluster B (*n* = 147)	Cluster C (*n* = 112)	*p*
Feto‐placental data at diagnosis				
Estimated foetal weight, g	1489 ± 590	2079 ± 582	2529 ± 622	< 0.01
Estimated foetal weight centile	12.8 [0.1–18]	28.7 [5–45.7]	68.0 [51.7–91.7]	< 0.01
Prenatal SGA^a^	159 (64.1)	59 (35.5)	6 (4.9)	< 0.01
Uterine arteries mean PI > 95th centile	113 (45.8)	30 (18)	8 (6.6)	< 0.01
Umbilical artery PI > 95th centile	58 (23)	8 (5)	5 (5)	< 0.01
Middle cerebral artery PI < 5th centile	66 (26)	8 (5)	5 (5)	< 0.01
Cerebroplacental ratio < 5th centile	92 (37)	18 (11)	7 (6)	< 0.01
Ductus venosus PI > 95th centile	20 (8)	4 (2.4)	2 (2)	< 0.01
Delivery data				
Gestational age at delivery, weeks	33.1 ± 3.3	37.0 ± 2.1	38.2 ± 1.5	< 0.01
Induction/planned delivery	234 (95)	152 (93)	111 (92)	0.40
Emergency caesarean section	93 (33.7)	32 (19)	19 (20)	< 0.01
Neonatal data				
Female sex	100 (40.3)	83 (50.0)	64 (52.3)	0.11
Birthweight, g	1551 ± 601	2368 ± 656	3243 ± 426	< 0.01
Birthweight centile	4.9 [0.1–4.75]	15.5 [1–20]	67.9 [50–90]	< 0.01
SGA^b^	206 (83)	91 (54)	6 (4)	< 0.01
Severe SGA^c^	170 (68)	52 (31)	3 (2)	< 0.01
LGA^d^	0 (0)	1 (0)	22 (18)	< 0.01
Apgar at 5 min less than 7	15 (6)	3 (1)	2 (1)	0.03
Neonatal umbilical artery pH	7.20 ± 0.08	7.22 ± 0.08	7.22 ± 0.10	0.17
Neonatal umbilical artery pH < 7.1	25 (10)	14 (9)	10 (7)	0.61
Admission to NICU	61 (24)	25 (13)	3 (2)	< 0.01
Days at NICU	37.1 [15–52]	28.6 [11–22]	15 [4.5–21.5]	0.56
Perinatal mortality	20 (8)	7 (4)	2 (1)	0.03

*Note:* Data are presented as number (%) or mean ± standard deviation as appropriate.

Abbreviations: LGA, large for gestational age; NICU, neonatal intensive care unit; PI, pulsatility index; SGA, small for gestational age.

^a^ Prenatal SGA defined as estimated foetal weight below 10th centile.

^b^ SGA defined as birthweight below 10th centile.

^c^ Severe SGA defined as birthweight below 3rd centile.

^d^ LGA defined as birthweight above the 90th centile.

#### 3.1.3 Cluster C: Late Cardiometabolic Preeclampsia

Cluster C was characterised by late gestational age at diagnosis, the highest maternal BMI and birthweight centile and the lowest values of sFlt1/PlGF ratio and uterine artery Doppler (Figure 1B). As compared to the other clusters, Cluster C contained the lowest rate of smoking (4%) and the highest rate of maternal comorbidities (61%), including 31% obesity, 15% pregestational diabetes and 12% autoimmune disorders. It also showed the lowest values of maternal sFlt1/PlGF, liver enzymes, urinary albumin/creatinine and rates of maternal severe complications (4%) with no cases of HELLP syndrome. Mean gestational age at diagnosis and delivery of Cluster C were 37 and 38 weeks, respectively. Cluster C exhibited the most favourable neonatal results, with the lowest rates of SGA (4%), neonatal complications (9%), NICU admissions (2%) and perinatal mortality (1%) (Table 2 and Figure 3). Actually, 18% of the neonates within this cluster were LGA.

## 4 Discussion

### 4.1 Main Findings

In this study, we identified three phenotypes of preeclampsia using an unsupervised ML which provided a data‐driven quantitative approach to classifying preeclampsia based on integrating clinical, laboratory and ultrasonographic variables. The three phenotypes identified correspond to early, late placental and late cardiometabolic preeclampsia, exhibit differential patterns of clinical presentation and resulting health complications for both the mother and the neonate. The classification proposed illustrates the potential of ML approaches to go beyond current terminologies based on the severity of the disease and support their role as a necessary step for the development of more effective strategies for screening, prevention and treatment.

The results of this study align with the conceptual framework that recognises preeclampsia as a heterogeneous disorder with multiple phenotypes. The Cluster A was characterised by features of defective placental development and severe hypertension. This group corresponds mainly to what has been long and extensively described as early preeclampsia [10, 12, 17]. Early preeclampsia associates with angiogenic imbalance, placental dysfunction and adverse maternal and neonatal complications, including foetal growth restriction [3, 25]. Notably, maternal previous preeclampsia and chronic hypertension seemed to be significant risk factors for Cluster A preeclampsia, suggesting that this phenotype is driven by a combination of maternal vascular dysfunction and abnormal placental development. This pathophysiology underpins current early preeclampsia prediction models based on first‐trimester markers of placental and angiogenic dysfunction and maternal blood pressure [47, 48] and prevention by aspirin [48].

An additional insight contribution of the present study is the distinction between two late‐onset phenotypes of preeclampsia. Cluster B included women who developed late preeclampsia with significant angiogenic imbalance and abnormal feto‐placental Doppler, suggesting a placental‐driven pathophysiology, with a less dominant vascular component. The findings support that this phenotype might represent a milder or later form of placental dysfunction, potentially serving as the main trigger for preeclampsia in these cases. Unlike other pheno‐groups, Cluster C was primarily associated with maternal cardiometabolic factors, including pregestational diabetes, obesity and other comorbidities, supporting maternal cardiometabolic predisposition as a major contributor in disease development. Indeed, previous studies have extensively highlighted that suboptimal pregestational cardiac performance or excessive pregnancy‐related cardiovascular demand may contribute to the development of preeclampsia, particularly late in gestation [19, 20, 21, 25]. Interestingly, Cluster C was associated with LGA rather than SGA, which is also in line with previous observations reporting an increased incidence of LGA in late‐onset preeclampsia [19, 21, 49].

The results of this study confirm and expand previous evidence suggesting that preeclampsia could be stratified taking into consideration the pathways placental dysfunction and maternal cardiovascular maladaptation to pregnancy [21, 50, 51, 52]. Recent evidence demonstrates that preeclampsia with and without foetal growth restriction exhibit distinct hemodynamic profiles, distinguishing resistance‐dominant and volume‐dominant phenotypes [18]. Early‐onset cases are usually associated to foetal growth restriction and characterised by low cardiac output, high total vascular resistance and concentric cardiac remodelling, while late‐onset cases usually associate normal foetal growth, high cardiac output and eccentric hypertrophy in the absence of pressure overload [14, 20, 25, 53, 54]. This has led to suggest that late‐onset preeclampsia could be primarily driven by maternal cardiovascular maladaptation [14, 22, 25, 55, 56, 57]. In the present study, we introduce that not all women with late preeclampsia exhibit a homogeneous pathophysiological pattern. Instead, ML identified in Cluster B a relevant phenotype of late preeclampsia associated with placental dysfunction, which resembled the features of early‐onset preeclampsia with a milder intensity. These findings confirm the results of an earlier study from our group, which reported that late‐onset preeclampsia consisted of two distinct phenotypes: one with abnormal uterine artery Doppler and angiogenic factors similar to those observed in early‐onset preeclampsia and the other with normal uterine artery Doppler and angiogenic factors. Very similar results have been recently reported by Chaiworapongsa et al. [24] identifying two phenotypes of term preeclampsia based on the angiogenic imbalance. Although direct measurements of cardiac output were not available in our study, we hypothesise that the cardiometabolic profile observed in Cluster C may correspond to this group with no clear signs of placental dysfunction. This phenotype could be linked with the suboptimal cardiac adaptation to pregnancy described in late preeclampsia. These findings underscore the need for future studies to complement these observations and confirm the role of maternal cardiovascular function in a subset of the preeclampsia phenotypes.

### 4.2 Interpretation

The delineation of the three phenotypes identified in this study underscores the importance of tailored approaches to advance in the understating of preeclampsia from a research and clinical standpoints [58, 59, 60]. Phenotypic classification holds potential to enhance the accuracy and performance of predictive algorithms, by integrating different lines of evidence into a unified, more comprehensive description of the various phenotypes composing a clinical syndrome. A more accurate classification of late preeclampsia might unveil the effects of aspirin in the phenotype of cases associated with placental dysfunction. Considering the clinical and pathophysiological differences among the different phenotypes of preeclampsia, as well as the use of treatments targeting specific pathways (e.g., angiogenic imbalance, maternal cardiometabolic health) might yield better outcomes when properly customised [12, 59, 60]. For instance, Mediterranean diet has shown a positive effect in reducing the rate of late‐onset preeclampsia by about 40%, which might potentially benefit Cluster C [61]. Future research should increasingly considering these different preeclampsia phenotypes [62] to improve the interpretation of data, help identifying phenotypes of cases more suitable for each specific intervention and suggest new therapeutic strategies for preeclampsia.

### 4.3 Strengths and Limitations

The main strength of our study lies in its design that comprehensive explores data from a well‐characterised prospective cohort of preeclampsia. Our unsupervised approach strengthens current knowledge by offering a phenotyping for these pregnancies. Furthermore, we included a reliably large number of preeclampsia cases since it is much higher than the recommended ratio of 70 observations per input variable in cluster analysis [63]. However, this study has some limitations. First, the study was conducted at a single tertiary referral centre with a predominantly white Mediterranean population, which may limit generalizability to other settings and populations. Second, maternal echocardiographic data and placental pathology/weight were not available in this population, which could have provided further insights. Autoimmune diseases comprise a heterogeneous group; depending on the specific condition, they may be more closely associated with either placental development or maternal cardiometabolic health. However, in our cohort, these conditions were analysed as a single category due to their low individual prevalence, preventing a more granular subgroup analysis. Furthermore, we lack longitudinal data prior to the clinical presentation of the disease which would be valuable for a more comprehensive understanding of the early stages of preeclampsia. Although individual variables may span similar ranges across groups, this reflects the continuous nature of preeclampsia. The present analysis was not intended for classification or prediction, but rather to provide a holistic, multidimensional characterisation of phenotypic patterns. Finally, external validity of our results is warranted to strengthen the generalizability of our findings.

## 5 Conclusions

Our study identified three clinically recognisable preeclampsia phenotypes using unsupervised ML methods applied to a comprehensive set of clinical variables. These phenotypes demonstrated differences in clinical presentation and pregnancy complications, including the presence of two patterns among late‐onset disease. These findings support the heterogeneity of preeclampsia and illustrate how data‐driven approaches can reconstruct clinically meaningful phenotypes of the disorder. However, these results should be considered hypothesis‐generating. Future studies in larger and independent cohorts are warranted and may help deepen the results reported here. These studies may lead to more sophisticated and refined approaches to distinguish phenotypes in preeclampsia, potentially boosting real advances in prediction, prevention and management.

## Author Contributions

Ohad Houri, Lina Youssef and Francesca Crovetto conceived the study, accessed and verified the underlying data, performed the analyses, drafted the manuscript and approved the final version. Maria Borrell, Maddalena Crimella, Maria Giulia Ferrante, Rommy H. Novoa, Irene Casas, Noelia Encabo and Leticia Benítez collected data and verified outcomes. Marta Larroya, Anna Peguero, Eva Meler and Sara Castro‐Barquero collected data, verified clinical variables, interpreted results, reviewed the manuscript and approved the final version. Bart Bijnens provided methodological advice and interpretation. Francesc Figueras provided supervision and clinical interpretation. Eduard Gratacós, Gabriel Bernardino and Fàtima Crispi provided senior supervision, conceptual input, funding/resources, critical revision and final approval. All authors had full access to the data, contributed to data interpretation, revised the manuscript for important intellectual content and approved the final version for submission. All authors meet the BJOG authorship criteria.

## Funding

The project was partially funded by grants from Departament de Recerca i Universitats de la Generalitat de Catalunya 2021‐SGR‐01422; Instituto de Salud Carlos III (PI22/00684, PI22/00109, PI24/00127), co‐funded by the European Union; Fundació Mutua Madrileña (AP180722022, AP16002/2024) and Fundació La Marató de TV3 (202415‐30‐31 and 82/257). Lina Youssef was supported by the BITRECS fellowship programme ‘Biomedicine International training research programme for excellent clinician‐scientist’ by Barcelona Clinic Hospital and funded by the ‘la Caixa’ Foundation (code num. LCF/PR/SP23/52950012). Irene casas was supported from Instituto de Salud Carlos III (CM23/00118). Leticia Benitez was supported from Instituto de Salud Carlos III (CM21/00058). Sara Castro‐Barquero was supported from Fundación Alfonso Martín Escudero fellowship and the Instituto de Salud Carlos III (CD24/00244). Gabriel Bernardino was supported by the FSE+ and ‘Agencia Estatal de investigacion’ MICIU/AEI/10.13039/501100011033 (RYC2022‐035960‐I, PID2023‐149959OA‐100). Fàtima Crispi was supported by Hospita Clinic Barcelona (Intensificació Interna), Instituto de Salud Carlos III (INT25/00064) and Fundació Occident, Spain.

## Consent

The authors have nothing to report.

## Conflicts of Interest

The authors declare no conflicts of interest.

## Supporting information


**Table S1:** Maternal characteristics within the entire cohort of women with preeclampsia.
**Table S2:** Pregnancy characteristics within the entire cohort of women with preeclampsia.
**Table S3:** Maternal complications within the entire cohort of women with preeclampsia.
**Table S4:** Neonatal characteristics within the entire cohort of women with preeclampsia.
**Table S5:** Risk of maternal complications according to preeclampsia phenotypes.
**Table S6:** Risk of neonatal complications according to preeclampsia phenotypes.
**Table S7:** Standardised mean differences (SMD) of clinical and laboratory variables between data‐driven preeclampsia clusters.
**Figure S1:** Flowchart of the study population.
**Figure S2:** Line plot of standardised parameter profiles across each preeclampsia phenotypes.

## Data Availability

The data that support the findings of this study are available on request from the corresponding author. The data are not publicly available due to privacy or ethical restrictions.

## References

[1] L. A. Magee , M. A. Brown , D. R. Hall , et al., “The 2021 International Society for the Study of Hypertension in Pregnancy Classification, Diagnosis & Management Recommendations for International Practice,” Pregnancy Hypertens 27 (2022): 148–169, 10.1016/j.preghy.2021.09.008. 35066406

[2] M. Fishel Bartal and B. M. Sibai , “Eclampsia in the 21st Century,” American Journal of Obstetrics and Gynecology 226, no. 2 (2022): S1237–S1253, 10.1016/j.ajog.2020.09.037. 32980358

[3] L. A. Magee , K. H. Nicolaides , and P. Von Dadelszen , “Preeclampsia,” New England Journal of Medicine 386, no. 19 (2022): 1817–1832, 10.1056/NEJMra2109523. 35544388

[4] L. Say , D. Chou , A. Gemmill , et al., “Global Causes of Maternal Death: A WHO Systematic Analysis,” Lancet Global Health 2, no. 6 (2014): e323–e333, 10.1016/S2214-109X(14)70227-X. 25103301

[5] E. Dimitriadis , D. L. Rolnik , W. Zhou , et al., “Pre‐Eclampsia,” Nature Reviews Disease Primers 9, no. 1 (2023): 8, 10.1038/s41572-023-00417-6. 36797292

[6] S. C. Kane and F. Da Silva Costa , “Risk Factors for Pre‐Eclampsia: Received Wisdom Versus Reality,” BJOG: An International Journal of Obstetrics and Gynaecology 131, no. 1 (2024): 63, 10.1111/1471-0528.17311. 36210639

[7] H. Carr , K. Johansson , J. M. Snowden , et al., “Maternal and Interpregnancy Factors and Risks of First‐Time Preeclampsia in Second Pregnancy: A Swedish National Cohort Study,” BJOG: An International Journal of Obstetrics & Gynaecology 133, no. 1 (2026): 154–164, 10.1111/1471-0528.18348. 40888026 PMC12676204

[8] O. Erez , R. Romero , E. Jung , et al., “Preeclampsia and Eclampsia: The Conceptual Evolution of a Syndrome,” American Journal of Obstetrics and Gynecology 226, no. 2 (2022): S786–S803, 10.1016/j.ajog.2021.12.001. 35177220 PMC8941666

[9] L. C. Poon , A. Shennan , J. A. Hyett , et al., “The International Federation of Gynecology and Obstetrics (figo) Initiative on Pre‐Eclampsia: A Pragmatic Guide for First‐Trimester Screening and Prevention,” International Journal of Gynecology & Obstetrics 145, no. S1 (2019): 1–33, 10.1002/ijgo.12802. PMC6944283 31111484

[10] G. J. Burton , C. W. Redman , J. M. Roberts , and A. Moffett , “Pre‐Eclampsia: Pathophysiology and Clinical Implications,” BMJ 366 (2019): l2381, 10.1136/bmj.l2381. 31307997

[11] E. Jung , R. Romero , L. Yeo , et al., “The Etiology of Preeclampsia,” American Journal of Obstetrics and Gynecology 226, no. 2 (2022): S844–S866, 10.1016/j.ajog.2021.11.1356. 35177222 PMC8988238

[12] J. M. Roberts , J. W. Rich‐Edwards , T. F. McElrath , L. Garmire , L. Myatt , and for the Global Pregnancy Collaboration , “Subtypes of Preeclampsia: Recognition and Determining Clinical Usefulness,” Hypertension 77, no. 5 (2021): 1430–1441, 10.1161/HYPERTENSIONAHA.120.14781. 33775113 PMC8103569

[13] P. Von Dadelszen , L. A. Magee , and J. M. Roberts , “Subclassification of Preeclampsia,” Hypertension in Pregnancy 22, no. 2 (2003): 143–148, 10.1081/PRG-120021060. 12908998

[14] E. Kalafat and B. Thilaganathan , “Cardiovascular Origins of Preeclampsia,” Current Opinion in Obstetrics & Gynecology 29, no. 6 (2017): 383–389, 10.1097/GCO.0000000000000419. 28961633

[15] L. C. Poon , L. A. Magee , S. Verlohren , et al., “A Literature Review and Best Practice Advice for Second and Third Trimester Risk Stratification, Monitoring, and Management of Pre‐Eclampsia: Compiled by the Pregnancy and Non‐Communicable Diseases Committee of FIGO (The International Federation of Gynecology and Obstetrics),” International Journal of Gynecology & Obstetrics 154, no. S1 (2021): 3–31, 10.1002/ijgo.13763. 34327714 PMC9290930

[16] S. Yagel , S. M. Cohen , and D. Goldman‐Wohl , “An Integrated Model of Preeclampsia: A Multifaceted Syndrome of the Maternal Cardiovascular‐Placental‐Fetal Array,” American Journal of Obstetrics and Gynecology 226, no. 2 (2022): S963–S972, 10.1016/j.ajog.2020.10.023. 33712272

[17] Gestational Hypertension and Preeclampsia_ACOG Practice Bulletin, Number 222,” Obstetrics & Gynecology 135, no. 6 (2020): e237–e260. 32443079 10.1097/AOG.0000000000003891

[18] T. Stampalija , C. Lees , T. Ghi , et al., “ isuog Consensus Statement on Maternal Hemodynamic Assessment in Hypertensive Disorders of Pregnancy and Fetal Growth Restriction,” Ultrasound in Obstetrics & Gynecology 66, no. 5 (2025): 681–696, 10.1002/uog.70040. 40997762 PMC12579779

[19] G. Masini , L. F. Foo , J. Tay , et al., “Preeclampsia Has Two Phenotypes Which Require Different Treatment Strategies,” American Journal of Obstetrics and Gynecology 226, no. 2 (2022): S1006–S1018, 10.1016/j.ajog.2020.10.052. 34774281

[20] K. Melchiorre , V. Giorgione , and B. Thilaganathan , “The Placenta and Preeclampsia: Villain or Victim?,” American Journal of Obstetrics and Gynecology 226, no. 2 (2022): S954–S962, 10.1016/j.ajog.2020.10.024. 33771361

[21] J. Tay , L. Foo , G. Masini , et al., “Early and Late Preeclampsia Are Characterized by High Cardiac Output, but in the Presence of Fetal Growth Restriction, Cardiac Output Is Low: Insights From a Prospective Study,” American Journal of Obstetrics and Gynecology 218, no. 5 (2018): 517.e1–517.e12, 10.1016/j.ajog.2018.02.007. 29474844

[22] B. Thilaganathan and E. Kalafat , “Cardiovascular System in Preeclampsia and Beyond,” Hypertension 73, no. 3 (2019): 522–531, 10.1161/HYPERTENSIONAHA.118.11191. 30712425 PMC6380450

[23] F. Crispi , C. Domínguez , E. Llurba , P. Martín‐Gallán , L. Cabero , and E. Gratacós , “Placental Angiogenic Growth Factors and Uterine Artery Doppler Findings for Characterization of Different Subsets in Preeclampsia and in Isolated Intrauterine Growth Restriction,” American Journal of Obstetrics and Gynecology 195, no. 1 (2006): 201–207, 10.1016/j.ajog.2006.01.014. 16545329

[24] T. Chaiworapongsa , R. Romero , F. Gotsch , et al., “Preeclampsia at Term Can Be Classified Into 2 Clusters With Different Clinical Characteristics and Outcomes Based on Angiogenic Biomarkers in Maternal Blood,” American Journal of Obstetrics and Gynecology 228, no. 5 (2023): 569.e1–569.e24, 10.1016/j.ajog.2022.11.001. PMC10149598 36336082

[25] S. Yagel , S. M. Cohen , I. Admati , et al., “Expert Review: Preeclampsia Type I and Type II,” American Journal of Obstetrics & Gynecology. MFM 5, no. 12 (2023): 101203, 10.1016/j.ajogmf.2023.101203. 37871693

[26] T. J. Loftus , B. Shickel , J. A. Balch , et al., “Phenotype Clustering in Health Care: A Narrative Review for Clinicians,” Frontiers in Artificial Intelligence 5 (2022): 842306, 10.3389/frai.2022.842306. 36034597 PMC9411746

[27] A. L. Beam , J. M. Drazen , I. S. Kohane , T. Y. Leong , A. K. Manrai , and E. J. Rubin , “Artificial Intelligence in Medicine,” New England Journal of Medicine 388, no. 13 (2023): 1220–1221, 10.1056/NEJMe2206291. 36988598

[28] B. Wang , A. M. Mezlini , F. Demir , et al., “Similarity Network Fusion for Aggregating Data Types on a Genomic Scale,” Nature Methods 11, no. 3 (2014): 333–337, 10.1038/nmeth.2810. 24464287

[29] S. Sanchez‐Martinez , N. Duchateau , T. Erdei , A. G. Fraser , B. H. Bijnens , and G. Piella , “Characterization of Myocardial Motion Patterns by Unsupervised Multiple Kernel Learning,” Medical Image Analysis 35 (2017): 70–82, 10.1016/j.media.2016.06.007. 27322071

[30] S. Pai and G. D. Bader , “Patient Similarity Networks for Precision Medicine,” Journal of Molecular Biology 430, no. 18 (2018): 2924–2938, 10.1016/j.jmb.2018.05.037. 29860027 PMC6097926

[31] G. Jimenez‐Perez , F. Loncaric , P. Marti Castellote , et al., “Machine Learning‐Based Phenotyping and Risk Assessment of Hypertrophic Cardiomyopathy—Linking ECGs, Morphology and Genotypes,” European Heart Journal Cardiovascular Imaging 23, no. Supplement_1 (2022): jeab289.431, 10.1093/ehjci/jeab289.431.

[32] M. Cikes , S. Sanchez‐Martinez , B. Claggett , et al., “Machine Learning‐Based Phenogrouping in Heart Failure to Identify Responders to Cardiac Resynchronization Therapy,” European Journal of Heart Failure 21, no. 1 (2019): 74–85, 10.1002/ejhf.1333. 30328654

[33] M. Motwani , D. Dey , D. S. Berman , et al., “Machine Learning for Prediction of All‐Cause Mortality in Patients With Suspected Coronary Artery Disease: A 5‐Year Multicentre Prospective Registry Analysis,” European Heart Journal 38 (2016): ehw188, 10.1093/eurheartj/ehw188. PMC5897836 27252451

[34] P. Garcia‐Canadilla , S. Sanchez‐Martinez , F. Crispi , and B. Bijnens , “Machine Learning in Fetal Cardiology: What to Expect,” Fetal Diagnosis and Therapy 47, no. 5 (2020): 363–372, 10.1159/000505021. 31910421

[35] J. Miranda , C. Paules , G. Noell , et al., “Similarity Network Fusion to Identify Phenotypes of Small‐For‐Gestational‐Age Fetuses,” iScience 26, no. 9 (2023): 107620, 10.1016/j.isci.2023.107620. 37694157 PMC10485038

[36] B. Salvatori , S. Wegener , G. Kotzaeridi , et al., “Identification and Validation of Gestational Diabetes Subgroups by Data‐Driven Cluster Analysis,” Diabetologia 67, no. 8 (2024): 1552–1566, 10.1007/s00125-024-06184-7. 38801521 PMC11343786

[37] B. W. Eberhard , K. J. Gray , D. W. Bates , and V. P. Kovacheva , “Deep Survival Analysis for Interpretable Time‐Varying Prediction of Preeclampsia Risk,” Journal of Biomedical Informatics 156 (2024): 104688, 10.1016/j.jbi.2024.104688. 39002866 PMC11349290

[38] M. A. Brown , L. A. Magee , L. C. Kenny , et al., “Hypertensive Disorders of Pregnancy: ISSHP Classification, Diagnosis, and Management Recommendations for International Practice,” Hypertension 72, no. 1 (2018): 24–43, 10.1161/HYPERTENSIONAHA.117.10803. 29899139

[39] F. P. Hadlock , R. B. Harrist , R. S. Sharman , R. L. Deter , and S. K. Park , “Estimation of Fetal Weight With the Use of Head, Body, and Femur Measurements—A Prospective Study,” American Journal of Obstetrics and Gynecology 151, no. 3 (1985): 333–337, 10.1016/0002-9378(85)90298-4. 3881966

[40] F. Figueras , E. Meler , A. Iraola , et al., “Customized Birthweight Standards for a Spanish Population,” European Journal of Obstetrics, Gynecology, and Reproductive Biology 136, no. 1 (2008): 20–24, 10.1016/j.ejogrb.2006.12.015. 17287065

[41] A. Bhide , G. Acharya , A. Baschat , et al., “ isuog Practice Guidelines (Updated): Use of Doppler Velocimetry in Obstetrics,” Ultrasound in Obstetrics & Gynecology 58, no. 2 (2021): 331–339, 10.1002/uog.23698. 34278615

[42] D. Arduini and G. Rizzo , “Normal Values of Pulsatility Index Front Fetal Vessels: A Cross‐Sectional Study on 1556 Healthy Fetuses,” JPME 18, no. 3 (1990): 165–172, 10.1515/jpme.1990.18.3.165. 2200862

[43] O. Gómez , F. Figueras , S. Fernández , et al., “Reference Ranges for Uterine Artery Mean Pulsatility Index at 11–41 Weeks of Gestation,” Ultrasound in Obstetrics & Gynecology 32, no. 2 (2008): 128–132, 10.1002/uog.5315. 18457355

[44] A. A. Baschat and U. Gembruch , “The Cerebroplacental Doppler Ratio Revisited,” Ultrasound in Obstetrics & Gynecology 21, no. 2 (2003): 124–127, 10.1002/uog.20. 12601831

[45] L. McInnes , J. Healy , and J. Melville , “UMAP: Uniform Manifold Approximation and Projection for Dimension Reduction,” preprint, arXiv, 2020, cited March 10, 2025, http://arxiv.org/abs/1802.03426.

[46] P. J. Rousseeuw , “Silhouettes: A Graphical Aid to the Interpretation and Validation of Cluster Analysis,” Journal of Computational and Applied Mathematics 20 (1987): 53–65, 10.1016/0377-0427(87)90125-7.

[47] M. Y. Tan , A. Syngelaki , L. C. Poon , et al., “Screening for Pre‐Eclampsia by Maternal Factors and Biomarkers at 11–13 Weeks' Gestation,” Ultrasound in Obstetrics & Gynecology 52, no. 2 (2018): 186–195, 10.1002/uog.19112. 29896812

[48] D. L. Rolnik , D. Wright , L. C. Y. Poon , et al., “ASPRE Trial: Performance of Screening for Preterm Pre‐Eclampsia,” Ultrasound in Obstetrics & Gynecology 50, no. 4 (2017): 492–495, 10.1002/uog.18816. 28741785

[49] S. Verlohren , K. Melchiorre , A. Khalil , and B. Thilaganathan , “Uterine Artery Doppler, Birth Weight and Timing of Onset of Pre‐Eclampsia: Providing Insights Into the Dual Etiology of Late‐Onset Pre‐Eclampsia: UtA Doppler, Birth Weight and Pre‐Eclampsia,” Ultrasound in Obstetrics & Gynecology 44, no. 3 (2014): 293–298, 10.1002/uog.13310. 24448891

[50] E. Ferrazzi , S. Zullino , T. Stampalija , et al., “Bedside Diagnosis of Two Major Clinical Phenotypes of Hypertensive Disorders of Pregnancy: Clinical Phenotypes of HDP,” Ultrasound in Obstetrics & Gynecology 48, no. 2 (2016): 224–231, 10.1002/uog.15741. 26350023

[51] E. Ferrazzi , T. Stampalija , L. Monasta , D. Di Martino , S. Vonck , and W. Gyselaers , “Maternal Hemodynamics: A Method to Classify Hypertensive Disorders of Pregnancy,” American Journal of Obstetrics and Gynecology 218, no. 1 (2018): 124.e1–124.e11, 10.1016/j.ajog.2017.10.226. 29102503

[52] W. Gyselaers , “Hemodynamic Pathways of Gestational Hypertension and Preeclampsia,” American Journal of Obstetrics and Gynecology 226, no. 2 (2022): S988–S1005, 10.1016/j.ajog.2021.11.022. 35177225

[53] H. Valensise , B. Vasapollo , G. Gagliardi , and G. P. Novelli , “Early and Late Preeclampsia: Two Different Maternal Hemodynamic States in the Latent Phase of the Disease,” Hypertension 52, no. 5 (2008): 873–880, 10.1161/HYPERTENSIONAHA.108.117358. 18824660

[54] K. Melchiorre , G. Sutherland , R. Sharma , M. Nanni , and B. Thilaganathan , “Mid‐Gestational Maternal Cardiovascular Profile in Preterm and Term Pre‐Eclampsia: A Prospective Study,” BJOG: An International Journal of Obstetrics & Gynaecology 120, no. 4 (2013): 496–504, 10.1111/1471-0528.12068. 23190437

[55] F. L. Foo , A. A. Mahendru , G. Masini , et al., “Association Between Prepregnancy Cardiovascular Function and Subsequent Preeclampsia or Fetal Growth Restriction,” Hypertension 72, no. 2 (2018): 442–450, 10.1161/HYPERTENSIONAHA.118.11092. 29967040

[56] B. S. Buddeberg , R. Sharma , J. M. O'Driscoll , A. Kaelin Agten , A. Khalil , and B. Thilaganathan , “Cardiac Maladaptation in Term Pregnancies With Preeclampsia,” Pregnancy Hypertens 13 (2018): 198–203, 10.1016/j.preghy.2018.06.015. 30177052

[57] C. Ghossein‐Doha , M. E. A. Spaanderman , R. Al Doulah , S. M. Van Kuijk , and L. L. H. Peeters , “Maternal Cardiac Adaptation to Subsequent Pregnancy in Formerly Pre‐Eclamptic Women According to Recurrence of Pre‐Eclampsia,” Ultrasound in Obstetrics & Gynecology 47, no. 1 (2016): 96–103, 10.1002/uog.15752. 26395883

[58] M. Ma'ayeh , K. M. Rood , D. Kniss , and M. M. Costantine , “Novel Interventions for the Prevention of Preeclampsia,” Current Hypertension Reports 22, no. 2 (2020): 17, 10.1007/s11906-020-1026-8. 32052203 PMC8237364

[59] L. Myatt , “The Prediction of Preeclampsia: The Way Forward,” American Journal of Obstetrics and Gynecology 226, no. 2 (2022): S1102–S1107.e8, 10.1016/j.ajog.2020.10.047. 33785181

[60] K. E. Duhig , P. T. Seed , A. Placzek , et al., “Prognostic Indicators of Severe Disease in Late Preterm Pre‐Eclampsia to Guide Decision Making on Timing of Delivery: The PEACOCK Study,” Pregnancy Hypertens 24 (2021): 90–95, 10.1016/j.preghy.2021.02.012. 33770588

[61] F. Crovetto , F. Crispi , R. Borras , et al., “Prevention of Late Preeclampsia With a Mediterranean Diet Intervention During Pregnancy,” 19th World Congress in Fetal Medicine.

[62] P. Parham and A. Moffett , “Variable NK Cell Receptors and Their MHC Class I Ligands in Immunity, Reproduction and Hsuman Evolution,” Nature Reviews. Immunology 13, no. 2 (2013): 133–144, 10.1038/nri3370. PMC3956658 23334245

[63] S. Dolnicar , B. Grün , F. Leisch , and K. Schmidt , “Required Sample Sizes for Data‐Driven Market Segmentation Analyses in Tourism,” Journal of Travel Research 53, no. 3 (2014): 296–306, 10.1177/0047287513496475.

