# Supplementary material for: Phenotyping Preeclampsia Using Unsupervised Machine Learning: A Prospective Cohort Study
Source: BJOG. 2026 May 14;133(10):1926–36. doi: 10.1111/1471-0528.70262 (PMC13485454; doi:10.1111/1471-0528.70262)
Supplement: Supplementary file 1 [file BJO-133-1926-s001.docx]

**SUPPLEMENTARY MATERIAL**

**SUPPLEMENTARY METHODS**

**Definitions**

*Preeclampsia* was defined as the onset of hypertension after 20 weeks of gestation accompanied by proteinuria and/or end-organ damage^1,2^. *Hypertension* was defined as a systolic blood pressure of ≥140 mmHg and/or diastolic blood pressure of ≥90 mmHg, measured on two occasions, 4 hours apart. *Proteinuria* was defined as a urine protein level ≥300 mg in a 24-hour collection or a urine protein-to-creatinine ratio ≥0.3 in a random urine specimen.^2^ *Maternal end-organ damage* was defined as: acute kidney injury (creatinine ≥1 mg/dL); liver involvement (elevated transaminases, e.g. alanine aminotransferase or aspartate aminotransferase >40 IU/L) with or without right upper quadrant or epigastric abdominal pain; neurological complications (e.g. eclampsia, altered mental status, blindness, stroke, clonus, severe headaches, and persistent visual scotomata); or hematological complications (thrombocytopenia-platelet count <150000/μL, disseminated intravascular coagulation, hemolysis).^3^ *Feto-placental end-organ damage* was defined as abnormal uterine artery Doppler (mean pulsatility index above 95^th^ centile) and/or fetal growth restriction (birthweight below the 3rd centile, or birthweight below the 10th centile together with abnormal cerebroplacental ratio or uterine artery Doppler).^1^

***Autoimmune diseases* included rheumatoid arthritis, autoimmune hepatitis, autoimmune hemolytic anemia, systemic lupus erythematosus, Raynaud phenomenon, granulomatosis with polyangiitis, essential thrombocytopenia, mixed connective tissue disease, Sjögren’s syndrome, scleroderma, and antiphospholipid syndrome.**

*Low socioeconomic status* was defined as women with no formal education beyond primary school and/or reported unemployment.

***Severe maternal complications* were defined as the presence of any of the following: (i) HELLP syndrome (lactate dehydrogenase >700 IU/L, Aspartate Aminotransferase to twice normal values and platelet count <100x109/L); (ii) Central nervous system dysfunction (eclampsia, stroke, reversible ischemic neurological deficit or cortical blindness); (iii) renal dysfunction (dialysis, serum creatinine concentration greater than 150 µmol/L, or urine output <0.5 mL/kg/h during 12 hours, according to renal insufficiency by RIFLE criteria**^4^**; or need for treatment with furosemide to maintain urine output >0.5 mL/kg/h for 3 hours); (v) pulmonary edema; (vi) a requirement for transfusion of blood products red cells; or (vii) maternal death.**

***Severe neonatal complications* were defined as the occurrence of any of the following: (i) need for resuscitation at birth (including stimulation, continuous positive airway pressure, positive pressure ventilation, cardiopulmonary resuscitation, or endotracheal intubation); (ii) or the presence of neonatal complications such as respiratory distress syndrome, pulmonary hypoplasia, necrotizing enterocolitis, neonatal infection, and brain intraventricular hemorrhage or death.**

**Clinical management of preeclamptic cases**

In cases of preeclampsia with severe features ^5^, pregnant women were hospitalized, and maternal blood pressure was monitored continuously, laboratory tests were assessed at least once a day, and fetal assessment was performed by daily cardiotocography and Doppler ultrasound at least twice a week. Magnesium sulfate for seizure prophylaxis was administered to all women. Management of hypertensive disorders during pregnancy in our cohort followed established international guidelines (ACOG/ISSHP), including recommended thresholds for treatment initiation and choice of first-line agents. Antihypertensive treatment was administered when blood pressure was persistently 160/110 mmHg or higher, with labetalol as a first-line drug. Indications for immediate delivery were uncontrollable blood pressure (systolic blood pressure ≥160 mm Hg or diastolic blood pressure ≥110 mm Hg not responsive to antihypertensive medication); persistent headaches refractory to treatment; epigastric pain or right upper pain unresponsive to repeat analgesics; visual disturbances, motor deficit or altered sensorium; stroke; myocardial infarction; renal dysfunction; pulmonary edema; eclampsia; HELLP syndrome suspected placental abruption and/or non-reassuring cardiotocographic reading.^2,5^ Elective delivery was performed beyond 34 weeks of gestation after completion of pulmonary maturation. In cases of preeclampsia without severe features, outpatient follow-up was mainly applied, and elective delivery was performed beyond 37 weeks of gestation.

**Machine learning clustering approach**

To explore potential subgroups within the preeclampsia cohort, we applied an unsupervised machine learning pipeline consisting of a first step of dimensionality reduction followed by a clustering algorithm. In contrast with supervised learning, which intends to develop predictive models of an independent variable (also known as label or outcome), unsupervised learning aims to identify natural groupings within a dataset without labels or outcome categories.

First, all variables included in the clustering analysis were standardized (z-score transformation) to ensure that differences in scale between variables did not disproportionately influence the analysis. Only continuous variables were included in the clustering analysis, as these methods rely on distance between values**.**

Because the dataset contained multiple correlated clinical and laboratory variables, we applied Uniform Manifold Approximation and Projection (UMAP) for dimensionality reduction. UMAP is a non-linear dimensionality technique, similar to Principal Components Analysis, that projects transform high-dimensional data into a lower-dimensional space while preserving the relative differences between observations. Each variable of the new space does not correspond uniquely o a single variable of the original one, but a combination of them; thus removing (non-linear) “correlations”. This step facilitates visualization of patient similarity patterns and improves clustering performance by reducing redunddancy and noise while maintaining the underlying structure of the data.

Dimensionality reduction was performed using Uniform Manifold Approximation and Projection (UMAP) with 5 components, a random state of 42, and single-threaded computation, while other parameters were kept at default settings. The resulting embedding was clustered using K-means with three clusters and a random state of 1.

To assess robustness, two complementary analyses were performed. First, sensitivity analyses were conducted by varying key UMAP hyperparameters, including the number of neighbors (10, 15, and 20), minimum distance (0.01, 0.1, and 0.5), and spread parameter (0.75, 1.0, and 1.25). Second, the clustering procedure was repeated 10 times on random 95% subsamples of the dataset.

Across all analyses, cluster structure and clinical profiles remained consistent, supporting the robustness of the identified phenotypes.

After dimensionality reduction, patient groups were identified using the k-means clustering algorithm. K-means is an unsupervised learning method that partitions individuals into a predefined number of groups (clusters) such that patients within the same cluster are more similar to each other than to patients in other clusters. The algorithm iteratively assigns individuals to clusters and updates cluster centers until the optimal grouping structure is achieved.

The optimal number of clusters was determined using the silhouette coefficient, a metric that evaluates how well clusters minimize variability within the cluster compared with differences other clusters. Higher silhouette values indicate better separation between clusters. We tested different numbers of clusters, between X and 10, and kept the number of clusters maximizing the silhouette coefficient.

To evaluate the robustness of the clustering solution, the dimensionality reduction and clustering process was repeated multiple times using different random seeds. The clustering results remained highly consistent across runs, indicating good stability of the identified groups.

Following identification of clusters, classical statistical analyses were performed to compare maternal characteristics, pregnancy features, and maternal and neonatal outcomes across the data-driven phenotypes**.**

**SUPPLEMENTARY RESULTS**

**Table S1.** Maternal characteristics within the entire cohort of women with preeclampsia.

|  | Total (N=482) |
| --- | --- |
| Maternal Age, years | 34.39 ± 5.57 |
| Ethnic Origin (Self-reported race or Ethnicity) |  |
| White | 314 (65.1%) |
| Hispanic | 121 (25.1%) |
| Asian | 44 (9.1%) |
| Low socioeconomic status^λ^ | 186 (38.6%) |
| Weight, Kg | 66.87 ± 13.4 |
| Pregestational Body Mass index, Kg/m^2^ | 25.54 ± 4.8 |
| Comorbidities | 192 (39.8%) |
| Obesity^Σ^ | 121 (25.1%) |
| Chronic hypertension | 74 (15.4%) |
| Pregestational Diabetes Mellitus | 30 (6.2%) |
| Autoimmune diseases | 31 (6.4%) |
| Renal Disease | 16 (3.3%) |
| Previous preeclampsia | 82 (17.0%) |
| Smoking | 57 (11.8%) |
| Nulliparity | 361 (74.9%) |
| Use of Assisted Reproductive Technologies | 91 (18.9%) |
| Data are presented as number (%) or mean ± standard deviation as appropriate  ^λ^Low socioeconomic status defined as no formal education beyond primary school and/or reported unemployment  ^Σ^Obesity defined as body mass index above 30 | |

**Table S2.** Pregnancy characteristics within the entire cohort of women with preeclampsia.

|  | Total (N=482) |
| --- | --- |
| *Maternal first trimester data* | |
| Systolic blood pressure, mmHg | 119.84 ± 12.17 |
| Diastolic blood pressure, mmHg | 77.34 ± 9.78 |
| Uterine arteries mean PI | 1.26 ± 0.55 |
| *Maternal data at diagnosis* | |
| Gestational age at diagnosis, weeks | 35.49 ± 3.78 |
| Systolic blood pressure, mmHg | 150.34 ± 16.97 |
| Diastolic blood pressure, mmHg | 94.44 ± 10.5 |
| sFlt-1/PlGF | 1835 ± 3238 |
| Aspartate Aminotransferase, U/L | 35.01 [17-29] |
| Alanine Aminotransferase, U/L | 37.64 [15-30] |
| Uric Acid, mg/dL | 5.50 [4.6-6.3] |
| Lactate Dehydrogenase, U/L | 378[242-451] |
| Platelet count, x103/L | 195[144-257] |
| Urinary Albumin/Creatinine | 1982 [439-1930] |
| Use of antihypertensive drugs | 251 (52) |
| Use of magnesium sulfate | 260 (54) |
| Use of furosemide | 34 (7) |
| Administration of corticosteroids for fetal lung maturation | 193 (40.8) |
| *Feto-placental data at diagnosis* | |
| Estimated fetal weight, g | 1916 ± 742 |
| Estimated fetal weight centile | 30.5 [0.1-91.7] |
| Prenatal SGAφ | 224 (46.5) |
| Uterine arteries mean PI >95th centile | 151 (31.3) |
| Umbilical artery PI >95th centile | 71 (14.7) |
| Middle cerebral artery PI <5th centile | 79 (15) |
| Cerebroplacental ratio <5th centile | 117 (21.7) |
| Ductus venosus PI >95th centile | 26 (4.8) |
| *Delivery data* | |
| Gestational age at delivery, weeks | 35.49 ± 2.8 |
| Induction/planned delivery | 445 (92) |
| Emergency cesarean section | 129 (27) |
| Data are presented as number (%), mean ± standard deviation or median [interquartile range]  sFlt1 denotes soluble fms-like tyrosine kinase 1; PlGF, placental growth factor; SGA, small for gestational age; PI, pulsatility index  φPrenatal SGA defined as estimated fetal weight below 10th centile. | |

**Table S3.** Maternal complications within the entire cohort of women with preeclampsia.

| Variable | Total (N=482) |
| --- | --- |
| Neurological prodrome | 92 (19.1%) |
| Kidney function impairment | 22 (4.5%) |
| Thrombocytopenia <100x10⁹/L | 44 (9.1%) |
| Impaired liver function | 44 (9.1%) |
| Pulmonary edema | 12 (2.4%) |
| Central Nervous System dysfunction | 3 (0.7%) |
| Preeclampsia with severe features | 193 (40.1%) |
| HELLP | 28 (5.8%) |
| Eclampsia | 2 (0.4%) |
| Postpartum preeclampsia | 23 (4.8%) |
| Gestational diabetes | 66 (13.6%) |
| Severe postpartum hemorrhage (blood products needed) | 6 (1.3%) |
| Maternal Death | 1 (0.2%) |
| Data are presented as number (%). | |

**Table S4.** Neonatal characteristics within the entire cohort of women with preeclampsia.

|  | Total (N=482) |
| --- | --- |
| Female sex | 247 (51.2) |
| Birthweight, grams | 2194 ± 892 |
| Birthweight centile | 22.8 ± 20.2 |
| SGAφ | 303 (62.8) |
| Severe SGAγ | 225 (46.6) |
| LGAη | 23 (4.7) |
| Apgar at 5 min less than 7 | 20 (4.1) |
| Neonatal Umbilical Artery pH <7.1 | 44 (10.1) |
| Admission to NICU | 80 (16.7) |
| Days at NICU | 34.55 ± 10.7 |
| Perinatal mortality | 26 (6) |
| Data are presented as number (%), or mean ± standard deviation.  SGA denotes Small for Gestational Age; LGA, Large for Gestational Age; NICU, Neonatal Intensive Care Unit.  φSGA defined as birthweight below 10th centile  γSevere SGA defined as birthweight below 3rd centile  ηLGA defined as birthweight above 90th centile. | |

**Table S5.** Risk of maternal complications according to preeclampsia phenotypes.

| **Variable** | **Cluster A** | **Cluster B** | **Cluster C** | **OR (A vs C)** | **95% CI** |
| --- | --- | --- | --- | --- | --- |
| HELLP syndrome | 20 (9%) | 4 (3%) | 1 (0.8%) | 11.1 | 1.4-85.5 |
| Central Nervous System dysfunction | 3 (1.2%) | 1 (0.7%) | 0 (0%) | 3.1 | 0.3-30.4 |
| Renal dysfunction | 18 (8%) | 1 (1%) | 1 (0.8%) | 9.9 | 1.3-74.8 |
| Pulmonary edema | 11 (5%) | 0 (0%) | 0 (0%) | 12.4 | 0.7-215 |
| Severe postpartum hemorrhage | 4 (2%) | 0 (0%) | 1 (0.8%) | 2.0 | 0.2-18.2 |
| Maternal death | 1 (0.4%) | 0 (0%) | 0 (0%) | 1.5 | 0.06-38 |
| Composite maternal complications | 52 (23%) | 8 (5%) | 4 (4%) | 7.6 | 2.6-21.7 |
| Data are numbers (percentage), and Odds Ratio (OR) with its 95% Confidence Interval (CI). | | | | | |

**Table S6.** Risk of neonatal complications according to preeclampsia phenotypes.

| **Variable** | **Cluster A** | **Cluster B** | **Cluster C** | **OR (A vs C)** | **95% CI** |
| --- | --- | --- | --- | --- | --- |
| Resuscitation at birth | 88 (39%) | 19 (13%) | 6 (6%) | 11.5 | 4.9-26.8 |
| Respiratory distress syndrome | 71 (32%) | 8 (5%) | 6 (6%) | 7.4 | 3.1-17.8 |
| Pulmonary hypoplasia | 2 (1%) | 0 (0%) | 0 (0%) | 2.5 | 0.12-53 |
| Necrotizing enterocolitis | 3 (1%) | 0 (0%) | 0 (0%) | 3.1 | 0.16-59 |
| Intraventricular hemorrhage | 14 (6%) | 1 (0.6%) | 0 (0%) | 15.7 | 0.9-270 |
| Neonatal infection | 21 (9.6%) | 0 (0%) | 1 (0.8%) | 11.8 | 1.6-88 |
| Composite neonatal complications | 91 (41%) | 22 (15%) | 10 (9%) | 7.1 | 3.4-14.9 |
| Data are numbers (percentage), and Odds Ratio (OR) with its 95% Confidence Interval (CI). | | | | | |

**Table S7.** Standardized Mean Differences (SMD) of Clinical and Laboratory Variables Between Data-Driven Preeclampsia Clusters

| **Variable** | **A vs B** | **A vs C** | **B vs C** |
| --- | --- | --- | --- |
| Body mass index | 0.06 | 1.791 | 1.732 |
| Maternal height | 0.07 | 0.259 | 0.19 |
| Maternal weight | 0.087 | 1.788 | 1.701 |
| First-trimester systolic blood pressure | 0.281 | 0.189 | 0.47 |
| First-trimester diastolic blood pressure | 0.104 | 0.213 | 0.317 |
| First-trimester mean arterial pressure | 0.192 | 0.226 | 0.418 |
| First-trimester- MAP pulsatility index | 0.342 | 0.109 | 0.232 |
| Gestational age at preeclampsia diagnosis | 0.58 | 0.195 | 0.386 |
| Mean arterial pressure at diagnosis | 1.407 | 0.546 | 0.862 |
| Systolic blood pressure at diagnosis | 1.399 | 0.462 | 0.936 |
| Diastolic blood pressure at diagnosis | 1.168 | 0.514 | 0.654 |
| sFlt-1 | 0.19 | 0.264 | 0.454 |
| PlGF | 0.41 | 0.074 | 0.483 |
| sFlt-1/PlGF ratio | 0.084 | 0.247 | 0.331 |
| Protein-to-creatinine ratio PE diagnosis | 0.762 | 0.521 | 0.24 |
| Serum creatinine at diagnosis | 0.253 | 0.241 | 0.012 |
| EFW at preeclampsia PE diagnosis | 0.543 | 0.413 | 0.129 |
| EFW percentile at PE diagnosis | 0.42 | 0.501 | 0.081 |
| GA at delivery | 0.659 | 0.317 | 0.341 |
| Neonatal birth weight | 0.636 | 0.482 | 0.154 |
| Birth weight percentile | 0.366 | 0.419 | 0.053 |
| Values represent standardized mean differences (SMDs) comparing clusters A, B, and C. EFW denotes estimated fetal weight; PlGF, placental growth factor; sFlt-1, soluble fms-like tyrosine kinase-1; MAP, mean arterial pressure; GA, gestational age. | | | |

**Figure S1.** Flowchart of the study population.

**Figure S2.** Line plot of standardized parameter profiles across each preeclampsia phenotypes.

Cluster A depicted in red, cluster B in violet, and cluster C in green. Each point represents the standardized mean value of each parameter within the preeclampsia clusters.

BMI denotes Body Mass Index; SES, Socioeconomic Status; ART, Assisted Reproductive Technologies; SBP, Systolic Blood Pressure; DBP, Diastolic Blood Pressure; MAP, Mean Arterial Pressure; sFlt1, Soluble Fms-like Tyrosine Kinase 1; PlGF, Placental Growth Factor; UA PI, Umbilical Artery Pulsatility Index; MCA PI, Middle Cerebral Artery Pulsatility Index; CPR, Cerebroplacental Ratio; DV PI, Ductus Venosus Pulsatility Index; Alb/Cr, Urinary Albumin/Creatinine Ratio; AST, Aspartate Aminotransferase; ALT, Alanine Aminotransferase; GGT, Gamma-Glutamyl Transferase; LDH, Lactate Dehydrogenase; HELLP, Hemolysis, Elevated Liver Enzymes, Low Platelets; SGA, Small for Gestational Age; NICU, Neonatal Intensive Care Unit.

**SUPPLEMENTARY** **REFERENCES**

1.	Magee LA, Brown MA, Hall DR, et al. The 2021 International Society for the Study of Hypertension in Pregnancy classification, diagnosis & management recommendations for international practice. *Pregnancy Hypertens*. 2022;27:148-169. doi:10.1016/j.preghy.2021.09.008

2.	Brown MA, Magee LA, Kenny LC, et al. Hypertensive Disorders of Pregnancy: ISSHP Classification, Diagnosis, and Management Recommendations for International Practice. *Hypertension*. 2018;72(1):24-43. doi:10.1161/HYPERTENSIONAHA.117.10803

3.	Poon LC, Shennan A, Hyett JA, et al. The International Federation of Gynecology and Obstetrics ( FIGO ) initiative on pre‐eclampsia: A pragmatic guide for first‐trimester screening and prevention. *Int J Gynecol Obstet*. 2019;145(S1):1-33. doi:10.1002/ijgo.12802

4.	Lopes JA, Jorge S. The RIFLE and AKIN classifications for acute kidney injury: a critical and comprehensive review. *Clin Kidney J*. 2013;6(1):8-14. doi:10.1093/ckj/sfs160

5.	Gestational Hypertension and Preeclampsia_ACOG Practice Bulletin, Number 222.
